# The superior colliculus motor region does not respond to finger tapping movements in humans

**DOI:** 10.1038/s41598-024-51835-9

**Published:** 2024-01-20

**Authors:** Nikhil G. Prabhu, Nicole Knodel, Marc Himmelbach

**Affiliations:** 1 Division of Neuropsychology, Center of Neurology, Hertie Institute for Clinical Brain Research, University of Tübingen, Tübingen, Germany; 2 Graduate Training Centre of Neuroscience, University of Tübingen, Tübingen, Germany; 3 International Max Planck Research School in Cognitive and Systems Neuroscience, University of Tübingen, Tübingen, Germany; 4 High Field Magnetic Resonance, Max Planck Institute for Biological Cybernetics, Tübingen, Germany

**Keywords:** Motor control, Sensorimotor processing, Superior colliculus

## Abstract

Electrophysiological studies in macaques and functional neuroimaging in humans revealed a motor region in the superior colliculus (SC) for upper limb reaching movements. Connectivity studies in macaques reported direct connections between this SC motor region and cortical premotor arm, hand, and finger regions. These findings motivated us to investigate if the human SC is also involved in sequential finger tapping movements. We analyzed fMRI task data of 130 subjects executing finger tapping from the Human Connectome Project. While we found strong signals in the SC for visual cues, we found no signals related to simple finger tapping. In subsequent experimental measurements, we searched for responses in the SC corresponding to complex above simple finger tapping sequences. We observed expected signal increases in cortical motor and premotor regions for complex compared to simple finger tapping, but no signal increases in the motor region of the SC. Despite evidence for direct anatomical connections of the SC motor region and cortical premotor hand and finger areas in macaques, our results suggest that the SC is not involved in simple or complex finger tapping in humans.

## Introduction

Several electrophysiological studies in macaques demonstrated the involvement of the SC in visually guided arm movements^1–5^. Philipp and Hoffmann^6^ reported the initiation and execution of arm movements upon electrical microstimulation of the SC in macaques. Two fMRI studies on visually guided reaching and a recent study on reaching to tactile targets confirmed these observations^7–9^. In macaques, Nagy et al.^10^ observed single cell activity in the SC in correlation with pushing a button with the index finger after and before a reaching movement. The same neurons were silent during reaching movements preceding and following button pushing. In humans, Prabhu and Himmelbach observed signal increases in the SC limb motor region for infrequent finger button presses in the abovementioned study on reaching to tactile targets^9^. In a control condition of this experiment, the subjects responded to visual oddball stimuli with a single button press but, in contrast to the paradigm reported by Nagy et al.^10^, without reaching movements (please refer to Prabhu and Himmelbach^9^ for more details). Anatomical studies in macaques reported connections between the upper limb motor region of the SC and the ventral premotor cortex (PMv^11–13^) and dorsal premotor cortex (PMd^11,13,14^). The involvement of the ventral and dorsal premotor cortex in the execution of simple and complex finger tapping movements has been shown repeatedly in human fMRI studies^15–19^ (please refer to Witt et al.^20^ for a meta-analysis of 38 studies). Based on these observations in macaques and humans, we assumed that the human SC limb motor region is not only involved in reaching but also in the execution of isolated finger movements.

We addressed this assumption with two approaches. We drew from the publicly available Human Connectome Project dataset (HCP^21^) and analyzed data of the HCP motor task, which had been modified from a study by Buckner et al.^22^. The subjects executed right and left toe movements, right and left finger movements, and tongue movements. In the analysis of a large sample of 130 subjects, we found no evidence of SC involvement with simple finger tapping in the HCP data. fMRI studies on the premotor cortex reported higher BOLD signal estimates for complex finger tapping sequences in comparison to simple finger tapping (for an overview and meta-analysis, please see Witt et al.^20^). Considering this often-replicated signal increase for complex finger tapping sequences in the cortex, we hypothesized that the null finding for simple finger tapping in the HCP data analysis might be due to the use of simple finger tapping only. We continued our search with a dedicated fMRI experiment, including simple and complex finger tapping in response to visual and auditory pacing cues. We measured and analyzed four subjects, each repeating the experiment four times on four different days with an exceptionally large number of blocks in multiple single-case replications. We recruited subjects with earlier experience in fMRI measurements who were familiar with typical conditions and constraints of fMRI measurements. Measuring a large number of trials in a small number of participants allowed us to invest more time into familiarization with the setup and the tasks for each participant before the actual measurements. The large number of trials per individual improved the reliability of the within-subject effect estimates compared to usually much smaller trial numbers per individual in a typical fMRI group analysis. Similar small-N approaches have been used earlier in fMRI^23–30^. Please see Smith and Little^31^, Baker et al.^32^, and Ince et al.^33^ for recent discussions of and perspectives on small-N approaches.

## Results

### Human connectome project data

**Figure 1:**
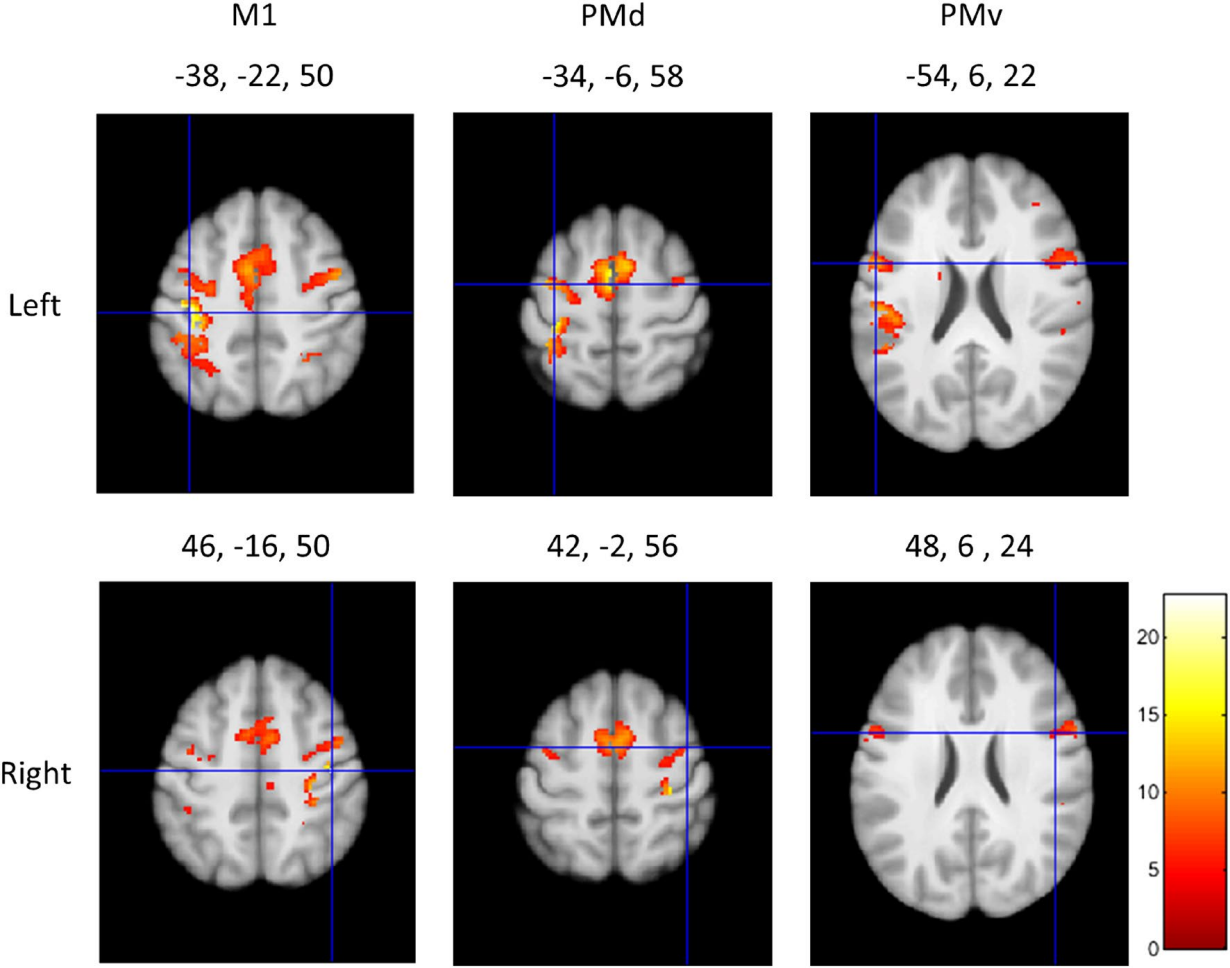
Cortical results for finger tapping from the HCP motor task dataset. Second-level GLM results centred on the peak coordinates in left M1, PMv, and PMd corresponding to right hand finger tapping (upper row), and right M1, PMv, and PMd corresponding to left hand finger tapping (lower row). The results have been thresholded at $p<0.05$ (FWE-corrected). The colourbar shows the range of t-values.

We first present the results of a second-level GLM analysis of right and left hand finger tapping from 130 subjects ($p<0.05$, FWE-corrected). The task consisted of 10 finger movements over 12 s, with 2 blocks of left hand and 2 blocks of right hand finger tapping per run and 2 runs per subject. Finger tapping conditions are correlated with robust activation clusters in the primary motor cortex (M1), dorsal pre-motor cortex (PMd), and ventral pre-motor cortex (PMv) (please see Witt et al.^20^ for a meta-analysis). The crosshairs in Fig. 1 are centered on the global maxima for left and right M1, PMd, and PMv corresponding to right and left hand finger tapping, respectively. These cortical results confirmed the validity of our approach and analysis for the analyses and results that follow for the SC motor region.

**Figure 2:**
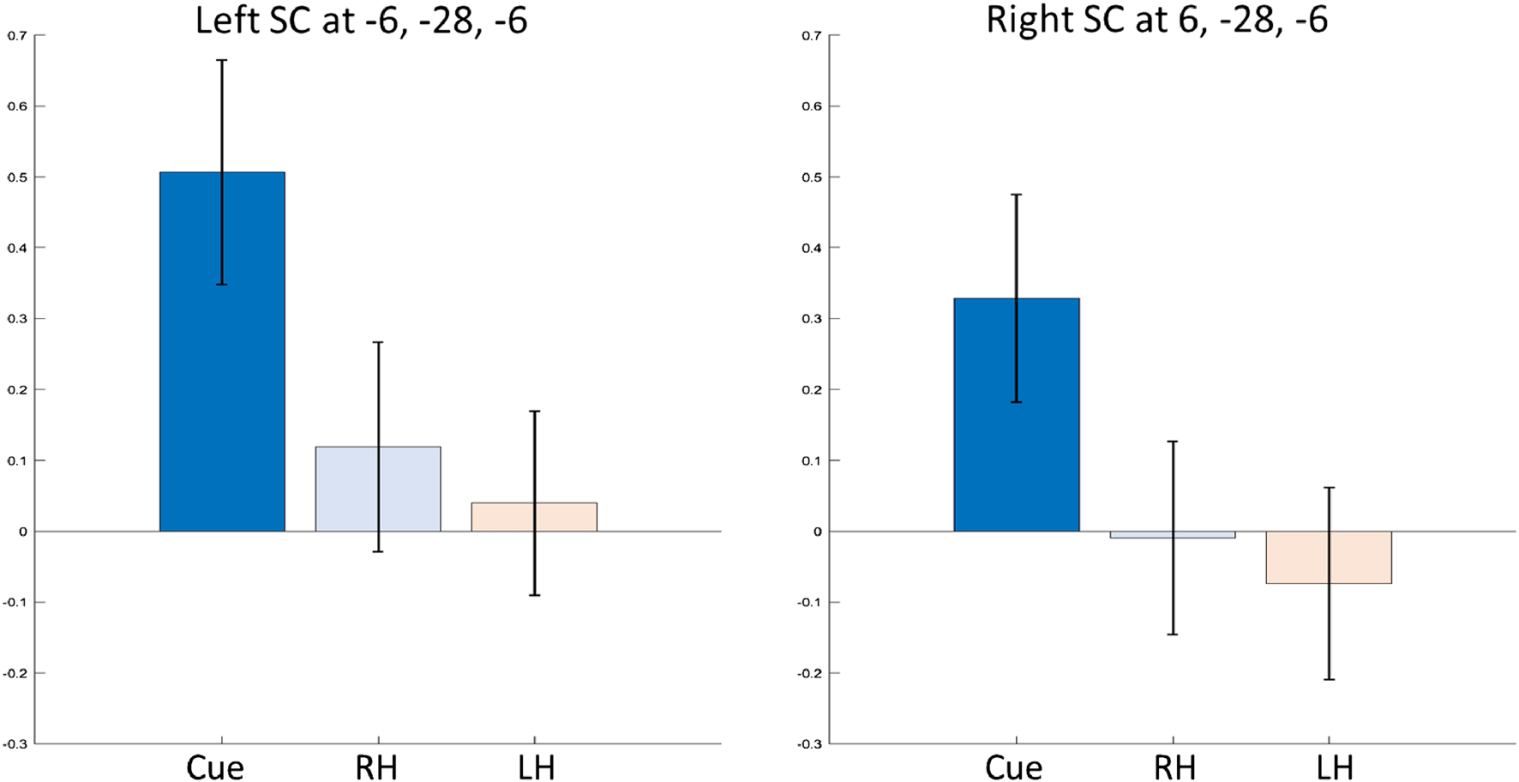
GLM contrast estimates at VOI locations from the HCP motor task dataset. Signal contributions from the three conditions—visual cue, right (RH) and left hand (LH) finger tapping at the VOI $-6, -28, -6$ and its corresponding location in the right SC: $6, -28, -6$. Error bars indicate 90% confidence intervals.

We analyzed GLM contrast estimates for visual cues, right hand, and left hand finger tapping at the location of button press activity that we observed in an earlier study^9^, i.e., at MNI coordinates $-6, -28, -6$ (volume of interest, VOI). We also included the corresponding location in the right SC at $6, -28, -6$ as the HCP dataset includes right and left hand finger tapping. We found a large, positive signal for the visual cues but no positive signal for right and left hand finger tapping. For the motor conditions, the 90% confidence intervals overlapped with 0 at both locations (Fig. 2). The figure shows 90% confidence intervals for a two-sided test. Please note that our hypothesis was uni-directional. Hence, we considered the results significant if the lower bound of the interval, which amounted to 95% confidence, was above zero.

**Figure 3:**
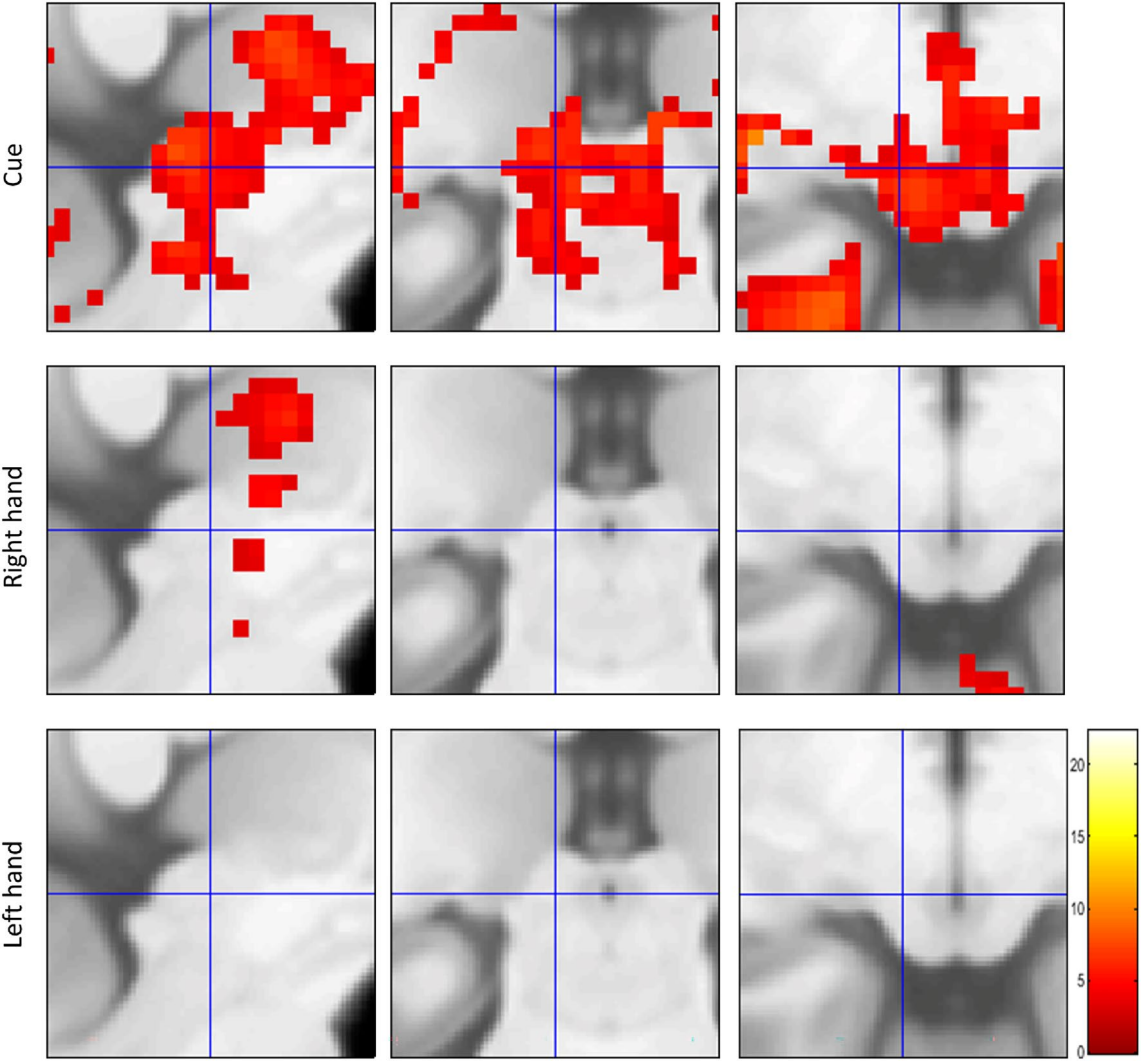
Voxel-wise GLM results in the SC from the HCP motor task dataset. Second-level GLM analysis results from cue, right hand, and left hand finger tapping conditions compared with fixation baseline ($p<0.001$, uncorrected). The images are centred on the VOI $-6, -28, -6$. There were no suprathreshold clusters in either left or right SC for the finger tapping conditions. The colourbar shows the range of t-values.

We further explored voxel-wise second-level GLM results for the three conditions of interest in the SC with a significance threshold of $p<0.001$, uncorrected. We observed a strong response for visual cues but found no response for the right- and left hand finger tapping movements in either the left or right SC (Fig. 3). These results confirmed and complemented the findings from the localized contrast estimates reported above.

### Simple and complex finger tapping experiment

#### Behavioural data

We further explored the lack of finger movement signals in the SC in our analysis of the group HCP data with an experiment including simple and complex finger tapping paced by visual and auditory stimuli. The combination of the two experimental factors resulted in four experimental conditions: visual simple finger tapping (VS), auditory simple finger tapping (AS), and visual and auditory complex finger tapping (VC and AC). We measured four subjects, each repeating the experiment on four different days. We monitored gaze fixation and finger movements during the fMRI measurements with MRI-compatible cameras. Out of 55 intact and complete fMRI runs from the four subjects, eye videos from 44 runs were available for the behavioral analysis, 12, 10, 12, and 10 videos, respectively, from the four subjects (please see methods section below for details). Each run consisted of 16 experimental condition blocks with 40 button presses per block, resulting in 640 button presses per run. The average number of unwanted saccades per run, breaking gaze fixation, varied between 0 and 10 in subjects 1, 2 and 3. In the 4-th subject, the average number of unwanted saccades varied between 3 and 29. In runs with only simple button presses, saccades per run varied between 0 and 2, whereas in runs with complex button presses, saccades per run varied between 0 and 4 in the first 3 subjects. In the 4-th subject, in runs with simple button presses, the number of saccades per run varied between 7 and 29, whereas in runs with complex button presses, it varied between 24 and 28.

#### Bold fMRI results

**Figure 4:**
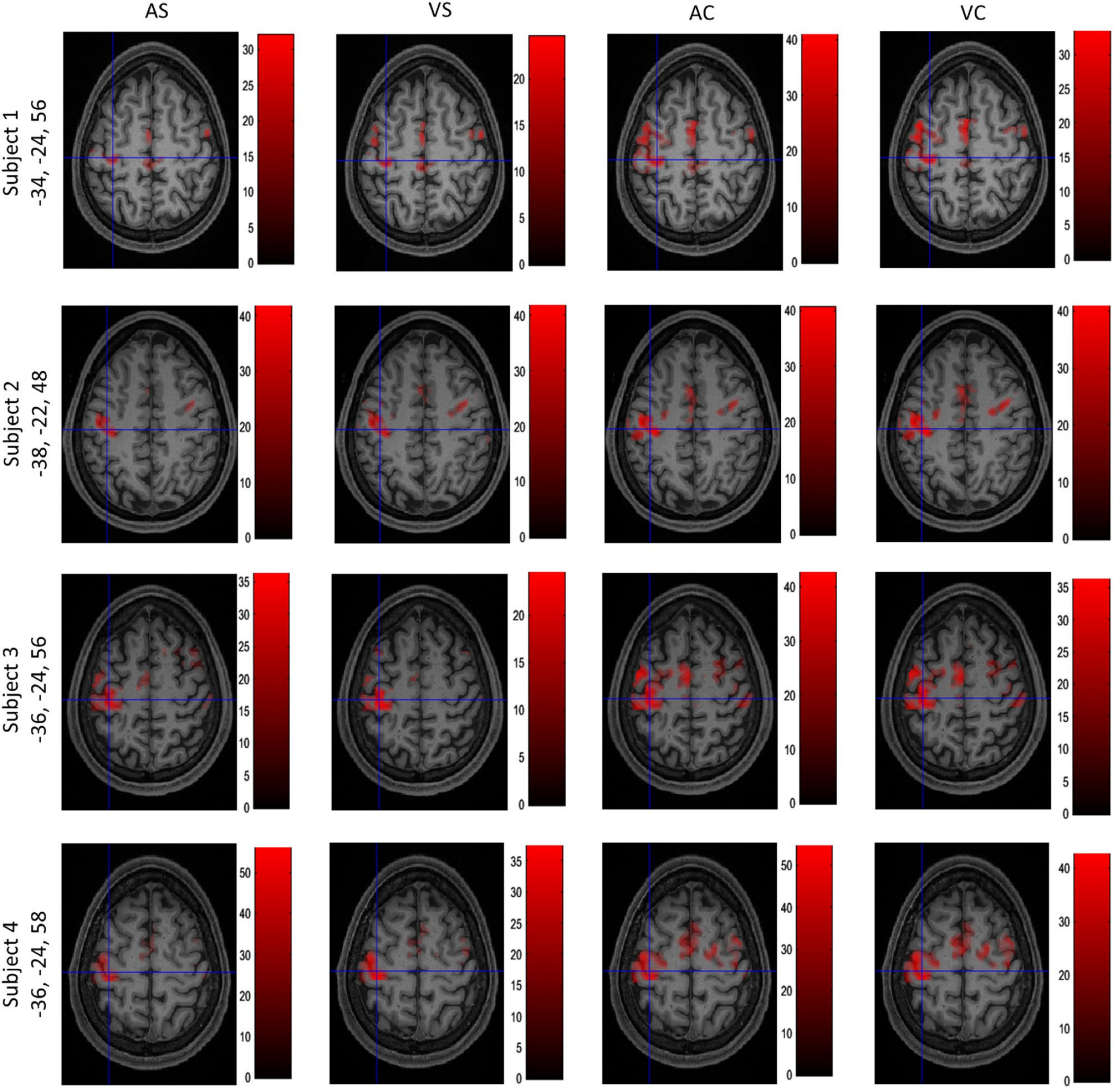
Local maxima in M1 in all subjects and conditions. Activation maps with crosshairs centred on the local maximum in the hand knob area of the left M1 across all four subjects. T-contrasts for the four experimental conditions against baseline: auditory simple (AS), auditory complex (AC), visual simple (VS), and visual complex (VC) thresholded at $p<0.05$, FWE-corrected. Colourbars show the range of t-values.

To ensure that the experimental paradigm elicited a response in cortical motor areas and thus confirm the validity of our approach, we first examined results from the hand knob in M1 (primary motor cortex), PMd (dorsal pre-motor cortex), and PMv (ventral pre-motor cortex). We visually localized the hand knob in M1 and identified the local maxima at this anatomical location. Figure 4 shows the coordinates for the local maxima in all four conditions at the hand knob in M1 for each subject thresholded at $p<0.05$, FWE-corrected. We searched for the local maxima close to the peak signal coordinates for PMd and PMv reported in the meta-analysis by Mayka et al.^35^. We then verified that these individual local maxima lay within the boundaries of the respective structures as described in the meta-analysis by Mayka et al.^34^. Local maxima for PMd and PMv across all conditions are reported in Table 1, thresholded at $p<0.05$, FWE-corrected. We found a positive response in all four task conditions in M1, PMd, and PMv in each subject.

**Table 1:** MNI co-ordinates for local maxima in the left PMd and PMv across all four finger tapping conditions, auditory simple, auditory complex, visual simple and visual complex, thresholded at $p<0.05$, FWE-corrected. The respective range of t-values is reported across the 4 conditions.

Subject	PMd MNI x, y, z	t-values	PMv MNI x, y, z	t-values
1	$-38$, $-18$, 48	10.49–19.53	$-58$, 0, 32	14.39–27.12
2	$-36$, $-16$, 44	22.26–32.12	$-56$, 6, 28	7.14–16.92
3	$-36$, $-18$, 58	21.39–42.46	$-58$, 2, 22	10.22–24.48
4	$-38$, $-18$, 50	37.58–49.35	$-54$, 6, 22	19.22–32.80

**Figure 5:**
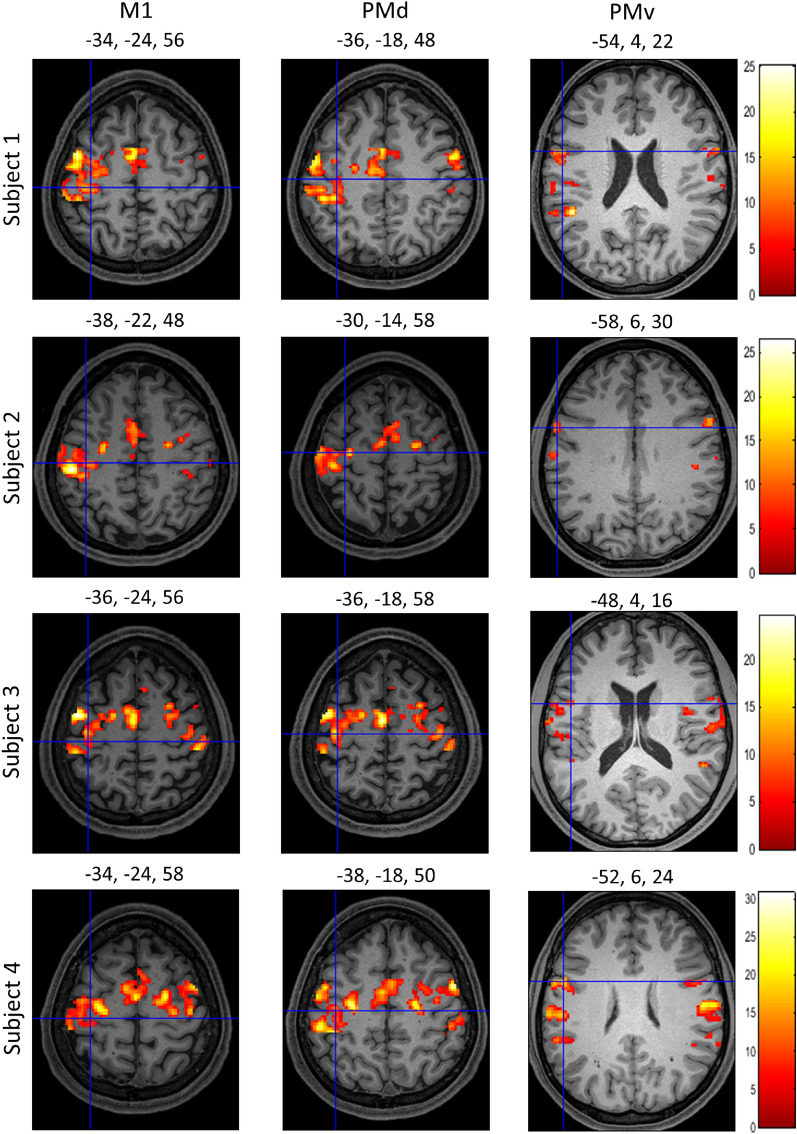
Signal increases in complex compared to simple finger tapping. Activation maps for a differential T-contrast with crosshairs centred on the local maxima in the left hand knob area of M1, PMd, and PMv in the 4 subjects ($p<0.05$, FWE-corrected). Colourbars show the range of t-values.

In the current experiment, we added a complex finger tapping paradigm that was not part of the HCP dataset because we expected a differential response compared to simple finger tapping. In agreement with our expectations and previous reports, we found a significant difference in M1, PMd, and PMv (Fig. 5). The local maxima reported in the figure were verified to lie within the boundaries of M1, PMd, and PMv as reported in the meta-analysis by Mayka et al.^34^.

**Figure 6:**
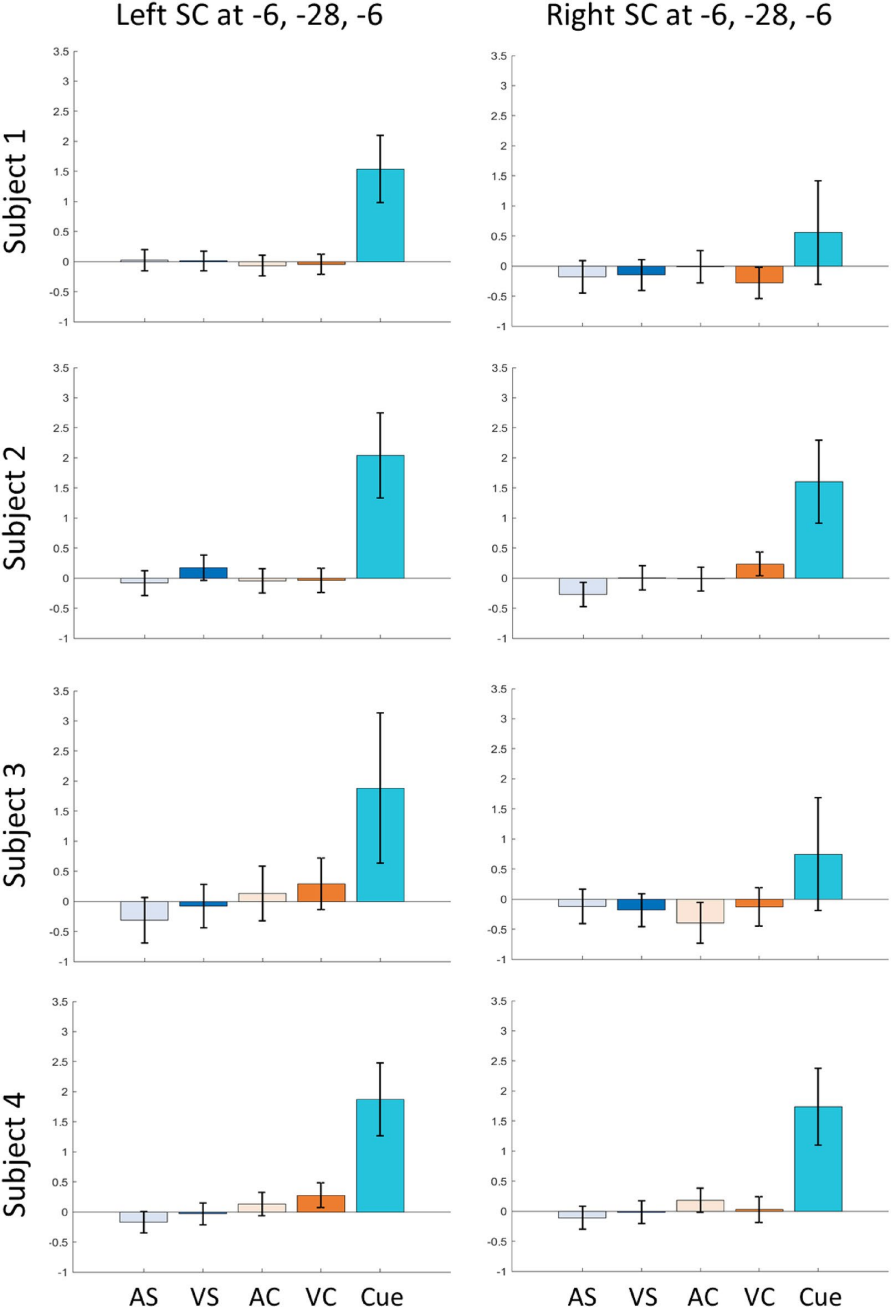
Contrast estimates at the a priori VOI in SC. GLM contrast estimates for the four tapping conditions and visual cues against baseline for each subject. *AS* auditory simple, *VS* visual simple, *AC* auditory complex, *VC* visual complex. The error bars indicate 90% confidence intervals.

We then analyzed GLM contrast estimates for each subject at the VOI $-6, -28, -6$ and in the corresponding location in the right SC for all four experimental conditions and visual cues (Fig. 6). Visual cues were associated with a significant positive signal increase in all four subjects, while none of the four finger tapping conditions showed a positive signal in any subject.

**Figure 7:**
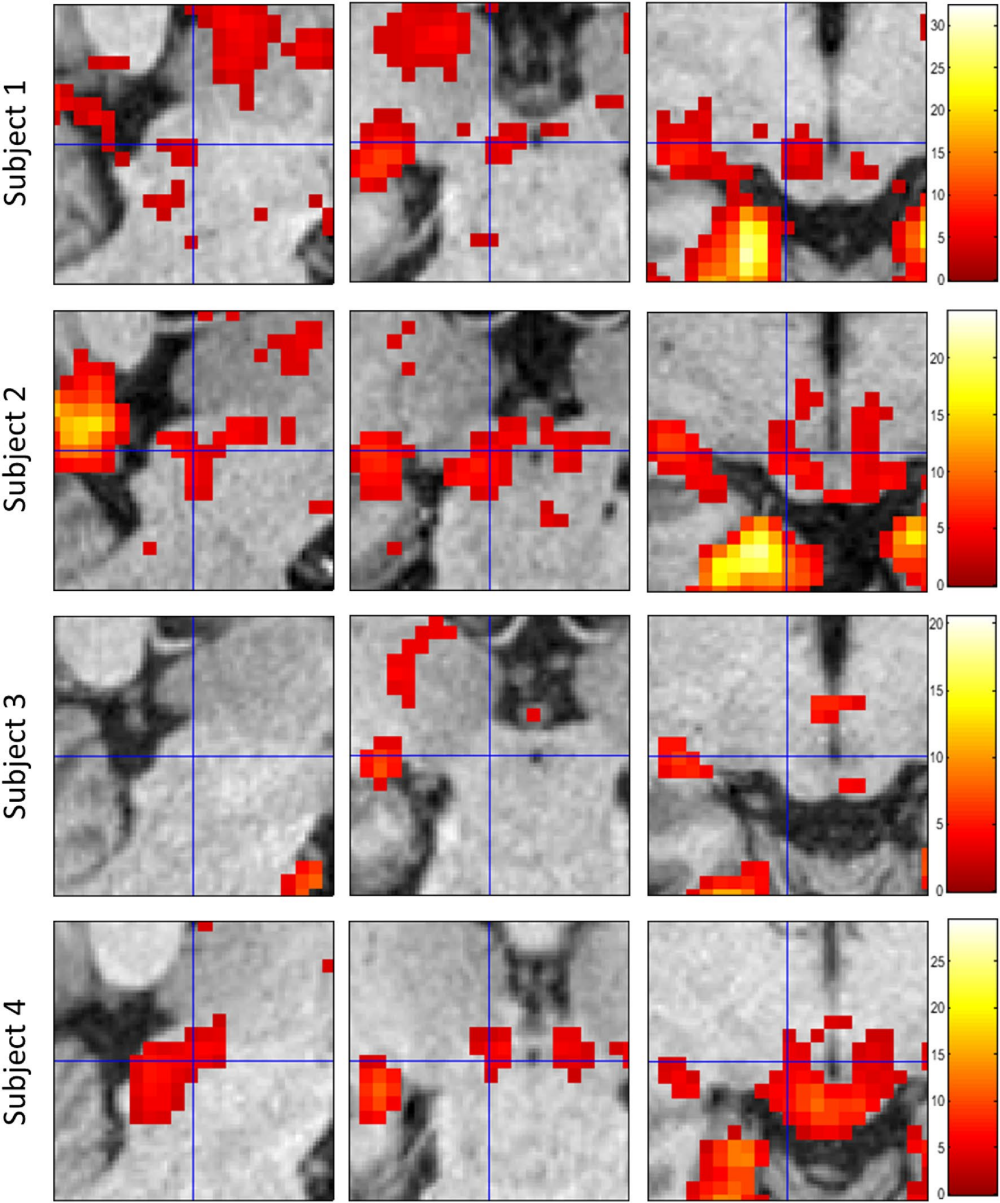
Voxel-wise GLM results for cues vs baseline. Suprathreshold voxels are shown in sagittal, coronal, and transverse sections of the SC centred on the VOI $-6, -28, -6$ at a threshold of $p<0.001$, uncorrected. Colourbars show the range of t-values.

We further explored voxel-wise GLM results for visual cues and the four conditions of interest in the SC with a significance threshold of $p<0.001$, uncorrected. A t-contrast against baseline for the cue condition revealed significant clusters in 3 out of 4 subjects (Fig. 7). We found no response in the SC above the threshold for any finger tapping condition in t-contrasts against the baseline. We further explored the finger tapping conditions using an F-contrast that would have revealed positive or negative signals in any of the four finger tapping conditions, again without positive results.

**Figure 8:**
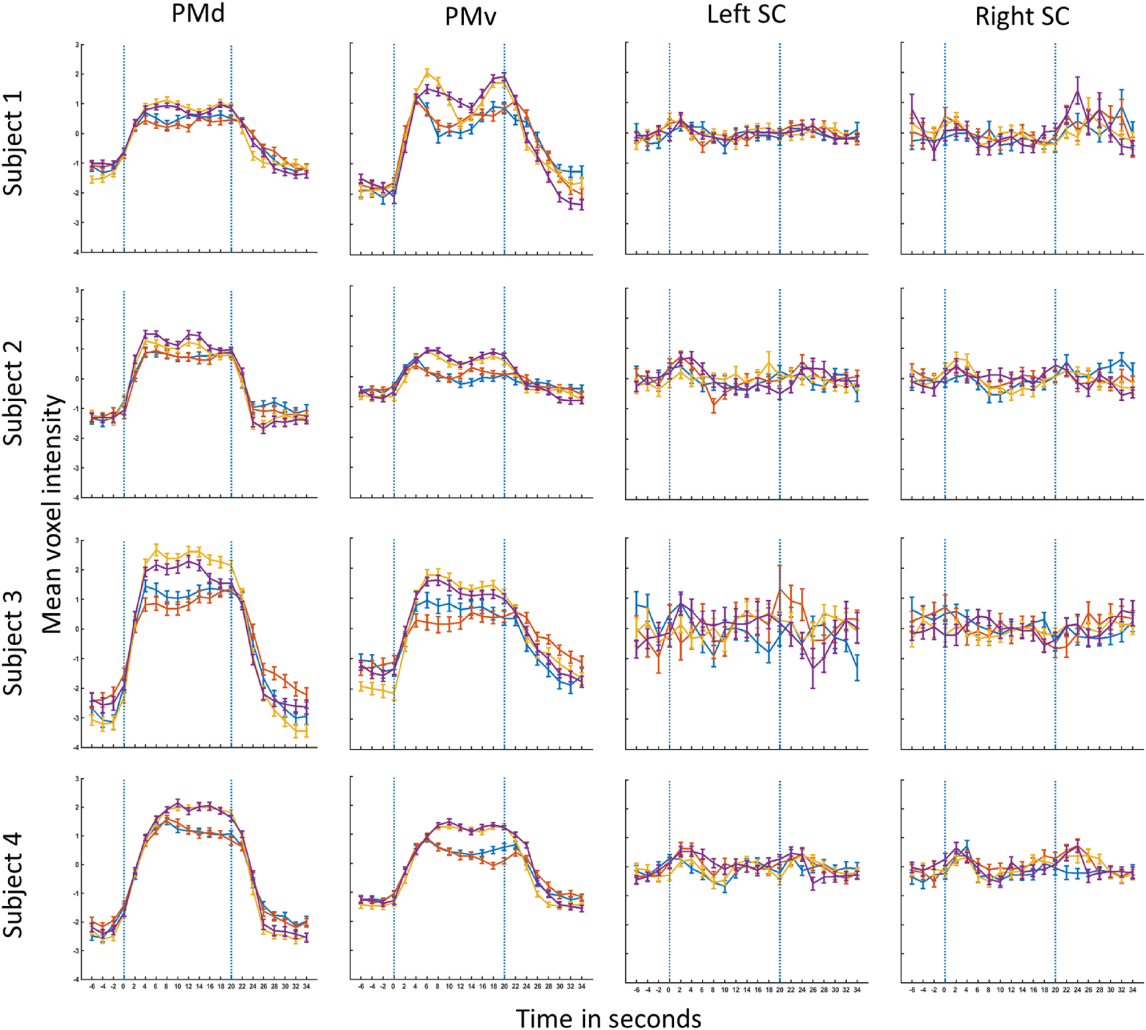
Signal time courses for each subject and condition in PMd, PMv, and SC. PMd and PMv time courses have been extracted for the voxels reported in Table 1. SC time courses have been extracted for the VOI $(-6, -28, -6)$ and its corresponding location in the right SC $(6, -28, -6)$. The signal time courses have been extracted for a time window from -6 s relative to block onset to 15 s after the end of the block. The two vertical blue dotted lines indicate the start and end of the block, 0 and 20 s, respectively. The error bars indicate standard error (SE). Blue—Auditory simple (AS), Red—Visual Simple (VS), Yellow—Auditory Complex (AC) and Purple—Visual Complex (VC).

We then examined signals in the four conditions in the SC, PMd, and PMv using detailed time courses (Fig. 8). We extracted the within-subject mean time courses in the SC for the a priori VOI for each subject. Timecourses from PMd and PMv were extracted for each subject’s individual PMd and PMv voxels as reported above (Table 1). PMd and PMv showed clear signal increases in good correspondence with block onset and duration (Fig. 8). We found the highest peaks above baseline in the PMd. We found higher peaks for complex tapping tasks than simple tapping in all 4 subjects in PMd and PMv. In stark contrast to time courses in PMd and PMv, there was no indication of any signal increase above baseline in the left or right SC.

## Discussion

Our analyses revealed no signals corresponding to finger tapping in the human SC. Given that Isa et al.^35^ and Cooper and McPeek^36^ implicated the SC in various motor tasks and activities across species and considering the sparsity of corresponding empirical data from humans, it is noteworthy that we found no evidence for a functional role of the human SC motor region in distal finger tapping movements.

The premotor cortices, PMd and PMv, have been implicated in the integration of sensory information towards the realization of sequential movements^15–19^ (please see Witt et al.^20^ for a meta-analysis). Studies by Fries^11^, Borra et al.^12^, Distler and Hoffmann^14^, and Fregosi and Rouiller^13^ described connections between the ventral and dorsal premotor cortices and the SC limb motor region. Results of our earlier study on tactile reaching movements included a control condition with button presses that showed a response in the SC motor region^9^. Together, the anatomical connections between the SC and cortical areas involved in the execution of sequential finger movements and the results from our previous study prompted us to investigate if the SC plays a role in finger tapping movements recorded by button presses. We first analyzed a large, publicly available dataset where we found activation clusters in M1, PMd, and PMv corresponding to finger tapping. We found no evidence of finger tapping related activity in the SC despite strong responses to visual stimulation in the same measurements. We then measured finger tapping activity in four subjects, with each subject repeating the experiment four times, where we saw activation in M1, PMv, and PMd corresponding to simple and complex finger-tapping sequences. Again, we found no signals in the SC corresponding to either simple or complex finger tapping sequences, although visual cues elicited a response similar to the results from our analysis of the HCP dataset. We replicated this pattern of results in all subjects with a large number of repetitions across different sessions and days.

Not only did the results of our earlier study in humans on tactile reaching clearly show a response in the SC for individual button presses, i.e., finger tapping with a button^9^, but also Nagy et al.^10^ reported neural activation in correlation with contact and with pushing of buttons in the macaque SC. This presents a contradiction which we aim to resolve with the following arguments. In contrast to the control condition in our earlier study on reaching, finger movements were repetitive and not individually instructed in our current study. The simple visual and auditory pacing stimuli in our current experimental measurements fundamentally differed from the odd-one-out stimuli in our previous experiment^9^ concerning their information value. It is also important to note the differences between our current study and the one by Nagy et al.^10^, with the paradigm in our study suited for discerning activity exclusive to finger tapping movements alone. Results from a study using electrical microstimulation in macaques might be relevant for our interpretation and understanding of these inconsistent results. Philipp and Hoffmann^6^ induced upper limb twitching, lifting, and extending movements by electrical stimulation of the SC upper limb motor region. Interestingly, the type of induced movements in the three animals depended on prior experience and training. The animal with extensive prior training in reaching paradigms showed the highest proportion of actual reaching movements upon electrical stimulation^6^. Considering these results, the SC limb motor region seems to not directly initiate movements of the arm, hand, or fingers but might be embedded in a more extensive network that gates and modulates their motor functions.

Finger tapping is a well-accepted paradigm in fMRI motor research with well-known and often-replicated cortical and subcortical responses. We used it to further delineate the functional contributions of the SC motor region in humans. Our results show that isolated, sequential finger tapping movements are not correlated with SC activity. This result does not mean that we falsified the involvement of the SC in any finger movements. A comparison between the paradigms and results of our current study and the two earlier positive reports^9,10^ indicates that the SC motor region might be involved in the execution of finger movements in a broader behavioral context with more ecological relevance.

While positive findings in fMRI are frequently confronted with suspicions of analytic flexibility^37,38^, negative findings in fMRI raise concerns about the sensitivity and detection power of the respective measurements and analyses. We are convinced that insufficient experimental methods or analyses cannot explain our negative findings. For the HCP data analysis and our own experimental measurements, activation clusters in M1, PMd, and PMv showed that our methods were adequate to reveal responses in motor areas corresponding to finger tapping. Activation clusters for the visual cue conditions from both analyses indicated that our methods were suited for signal detection at the SC. The HCP analysis showed that the SC does not play a role in executing simple finger tapping movements. With the second study, we replicated negative findings from the analysis of the HCP dataset for simple finger tapping in four individual subjects. In addition, we found that this result does not change with more complex but overlearned finger tapping sequences. The inspection of our descriptive data clearly showed an overlap of motor related signals at the SC with baseline signal levels in each analysis. While non-significant, inferential statistics cannot provide conclusive evidence for a null finding, the descriptive data from both analyses convinced us of a true null finding at the human SC motor region for isolated finger tapping movements.

We conclude that we have evidence against an involvement of the human SC motor region in isolated finger tapping movements. Our null results contrast with previous reports of signals in the human and macaque’s SC during button pushing with fingers. This apparent contradiction can be resolved by the assumption that the SC motor region might be involved in finger movements that are embedded in larger, ecologically more relevant behavioral contexts. Paradigms with finger movements in a behavioral context, such as object manipulation or grasping, should be compared to isolated finger movements in future studies.

## Methods

### Human connectome project data

#### Subject details

We analyzed 130 subjects from the HCP database with a complete dataset for the motor task (73 females and 57 males, age range 22–36 years, 10 left-handed and 120 right-handed) with normal or corrected-to-normal visual acuity.

#### Experimental setup and paradigm

The HCP motor task consisted of 13 blocks per run, 10 movement blocks, and 3 interspersed fixation blocks. Each movement block lasted 12 s and consisted of 10 movements. A movement block could consist of tapping the fingers of the left hand or the right hand, squeezing of the left or right toe, or a movement of the tongue depending on a visual cue that was shown for 3 s just before the start of the block. The 10 movement blocks were split into 2 tongue movement blocks, 4 hand movement blocks (2 right hand and 2 left hand) and 4 foot movement blocks (2 right foot and 2 left foot). The 3 fixation blocks in a run lasted 15 s in duration. 2 runs with 13 blocks each were measured from each subject^39^.

#### fMRI data acquisition

Data acquisition was performed at Washington University using a modified 3T Siemens Skyra MRI scanner. A 32-channel head coil was used for the whole-brain acquisition of functional measurements (Gradient echo-EPI) with a TR = 720 ms, TE = 33.1 ms, flip angle = 52$^{\circ }$, FOV = 208, 72 slices, with a voxel resolution of 2.0 mm isotropic, and a multi-band acceleration factor of 8. The two runs for the motor tasks were measured with opposing phase-encoding directions of right to left and left to right, respectively. The structural images were acquired with a 3D MPRAGE sequence TR = 2400 s, TE = 2.22 ms, flip angle = 8$^{\circ }$, FOV = 224 mm $\times$ 224 mm $(320 \times 320$ matrix), and a voxel resolution of 0.7 mm isotropic. For further details on the imaging protocols of the HCP, please refer to https://www.humanconnectome.org/hcp-protocols-ya-3t-imaging.

#### A priori definition of the volume of interest

In a preceding study on reaching-related activity in the SC, we observed BOLD activity with finger tapping for button presses in response to oddball stimuli that were shown in control conditions^9^. We extracted data from a $3 \times 3 \times 3$ grid of 27 voxels in the posterior-lateral SC limb motor region in the left and right SC. We then identified the voxel with the highest t-value in the finger tapping/button press condition and averaged these voxel coordinates across subjects. The resultant voxel was located at $-6, -28, -6$ (MNI coordinates; left SC). We used this voxel as the volume of interest for the analyses in the current study. In addition, we also examined results at the corresponding location in the right SC at $6, -28, -6$.

#### Data pre-processing

We used the minimally pre-processed dataset that was available with the HCP consortium. This data had already undergone pre-processing procedures like re-alignment, co-registration, and normalization except for smoothing (for details, refer to Glasser et al.^40^). We smoothed the data with a full-width half-maximum (FWHM) Gaussian kernel of 3.0 mm.

#### fMRI data analysis

We defined a first-level model with block onsets for the cue, left hand finger movements, right hand finger movements, left toe movements, right toe movements, and tongue movements with block durations of 3 s for the cues and 12 s for the movement conditions. These regressors were convolved with a modified hemodynamic response function with a shorter onset-to-peak time of 4 s that better fits neurovascular response dynamics in the SC^41^. The HCP data comprises 12 re-alignment parameters, out of which the first 6, corresponding to 3 translations and 3 rotations of head movements, were used as regressors of no interest. We then calculated contrast images with each condition in the experiment compared against the baseline. The second-level model was built with the contrasts of interest—cue, left hand, and right hand finger movements. We analyzed group GLM contrast estimates at the VOI mentioned above. We expected positive signal increases and considered results significant if the lower bound of the two-sided 90% confidence interval, which amounted to 95% confidence for our uni-directional hypothesis, did not include the zero baseline.

Additionally, we conducted a voxel-wise analysis of cortical regions and SC surrounding the VOI. Results were considered significant, surviving a voxel-level threshold of 0.05 FWE-corrected for cortical results and 0.001 uncorrected for results in the SC. Please note that the more conservative threshold for cortical data, in combination with the more lenient threshold for SC data, worked against our conclusion of a null finding at the SC with positive signal detection in cortical motor areas.

### Simple and complex finger tapping experiment

#### Subject details

We conducted the study using 3T BOLD fMRI with four right-handed healthy volunteers (2 females, 2 males, age range 25–32 years) with normal or corrected-to-normal visual acuity. Each subject performed the experiment four times, with one session comprising four runs per day. The experiments were conducted with the approval of the Ethics Committee at the Medical Faculty of the Eberhard-Karls-University and the University Hospital Tübingen in compliance with the ethical standards established by the 1964 declaration of Helsinki in its latest version. Informed consent was obtained from all subjects.

#### Experimental setup and paradigm

Subjects held a button box in their right hand with four buttons and wore headphones. We placed cushions (NoMoCo Pillow, Inc., La Jolla, USA) in the scanner’s head coil to immobilize the subjects’ heads. Two MR-compatible cameras (MRC systems GmbH, Germany) operating at 30 Hz, one to monitor finger movements and the other to monitor eye movements, were attached to a stand placed across the subject’s waist. Fibre optic cables carrying light from three LEDs: a white fixation/pacing LED, a green cue LED, and a red cue LED were attached to the uppermost central part of the stand. Subjects saw the optic fiber endings through a mirror attached to the head coil. Auditory pacing stimuli were delivered through the headphones. The experimental paradigm, including stimulus delivery and control of timing, was implemented on an ’MBED LPC 1768’ microcontroller (Arm Limited). All analyses were conducted using MATLAB (version R2018a, The MathWorks Inc., Natick, MA, USA).

We trained subjects to pace finger tapping following visual or auditory pacing stimuli just before the experiment began until they successfully matched two blocks of stimuli with finger tapping in a row. Our subjects accomplished this in a maximum of 10 min. Finger tapping could have been of two kinds—simple or complex finger tapping. Simple finger tapping involved pressing one of the four buttons paced by cues, while complex finger tapping involved repetitions of the four buttons: 2 times the first button (index finger), 4 times the second button (middle finger), 1 time the third button (ring finger), 3 times the fourth button (little finger) followed by finger tapping in the reverse order starting from the little finger (task adapted from Kuhtz-Buschbeck et al.^42^). The simple and complex finger tapping sequence blocks were informed by a cue of 1 s duration. A green LED cued the simple finger tapping block and a red LED cued the complex finger tapping block. For the simple finger tapping task, the green LED also cued the finger (any of the four) to be used in the impending block with the number of times it blinked, i.e., one blink for the first finger (index finger), two blinks for the second finger (middle finger) and so on. Block pacing stimuli were visual or auditory, presented at 2 Hz with either a blinking white LED or a ’beep’ delivered through headphones, respectively. The scanner room was darkened entirely by covering windows and displays with black opaque film. We set the brightness of the fixation light to a level just enough to discern it from the surroundings, ensuring that it did not illuminate the workspace. The subjects looked at the central fixation light throughout the experimental run time of 8 min and 50 s per run. The two modalities, visual and auditory, along with the two types of responses, simple and complex finger tapping, were combined in a $2 \times 2$ factorial design with 4 conditions: visual simple (VS), visual complex (VC), auditory simple (AS), and auditory complex (AC), two of them being part of each run, with one common factor. The four resulting runs, AS-VS, AC-VC, AS-AC, and VS-VC, were ordered in a Latin-square arrangement across the 16 runs within each subject to control for sequence effects. The two conditions in each run alternated as 20 s blocks of 40 trials, each experimental block interleaved by a fixation baseline of 15.5 s.

#### Analysis of saccade data

Eye videos were converted into eye traces with DeepLabCut^43^. Saccade data with a threshold of 2° visual angle were extracted from these eye traces. Synchronization errors in the video frame grabber meant several videos were unreliable for analysis. Missing frames in the videos did not allow for a temporal analysis of saccades in the video. Hence, we analyzed and reported the total number of saccades per subject per run.

#### fMRI data acquisition

We measured using a 3T Siemens Prisma scanner with a 64-channel head coil. 258 functional images (T2* weighted) were acquired as part of each run with an interleaved BOLD imaging of 28 slices in a coronal orientation covering the pre-motor areas and the brainstem, bilaterally. The images were acquired at a resolution of 2 mm isotropic with a TR = 2360 ms, TE = 35.0 ms, flip angle = 90$^{\circ }$, FOV = 180 mm $\times$ 180 mm ($90 \times 90$ matrix) and a phase encoding direction of foot to head. At the end of each imaging session, a whole-brain EPI image was acquired to aid in co-registration with the functional partial volumes. A structural image (T1 weighted) was acquired per subject using an MP-RAGE sequence with a resolution of 0.8mm isotropic and TR = 2400 ms, TE = 2.22 ms, flip angle: 8$^{\circ }$, FOV = 240 mm $\times$ 256 mm ($300 \times 320$ matrix).

#### Pre-processing of MRI data

Pre-processing and the following GLM analysis were carried out using Statistical Parametric Mapping (SPM12, Wellcome Trust Centre for Neuroimaging, London, UK), which was implemented in MATLAB (version R2018a, The MathWorks Inc., Natick, MA, USA). During an inspection of the images converted into the Nifti format, we observed that some runs had distorted images. Runs with image artifacts and runs with unreliable button press timings due to technical errors were discarded from further analysis. The number of runs that underwent further analysis were all 16 runs from the first subject, 12 runs from the second, 12 runs from the third, and 15 runs from the last subject (55 runs in total). We deleted the first five images from each run to allow scanner signals to reach a steady state. The rest of the images underwent a re-alignment procedure as defined in SPM, correcting for motion. The co-registration procedure was performed as follows. First, the mean EPI scans from each of the sessions in a subject were co-registered with the whole-brain EPI scan of the respective session. Next, the whole-brain EPI scans from all sessions of a subject were co-registered with the whole-brain EPI image from the first session of the subject. Lastly, the whole-brain EPI scan from the first session was co-registered with the structural image, followed by an inspection for conformity between the mean functional scans across sessions with the respective structural image. Mis-aligned images were manually oriented with the structural images before normalization with an MNI152 (Montreal Neurological Institute) template. This procedure resulted in good co-registration between the functional, structural, and template images. The images were then smoothed with a full-width half-maximum (FWHM) Gaussian kernel of 3 mm.

#### fMRI data analysis

A first-level analysis for each subject was performed using SPM12 with a dataset that included all sessions from different days. The first six motion parameters from the re-alignment step of the pre-processing stage were used as regressors of no interest. The first level model was designed with block onsets from each condition: Visual simple (VS), Visual complex (VC), Auditory simple (AS), and Auditory complex (AC), along with block durations and, onsets and durations of visual cues as regressors of interest. These regressors were convolved with a modified hemodynamic response function with a shorter onset-to-peak time of 4s that better fits neurovascular response dynamics in the SC than the canonical response function implemented in SPM12^41^. Contrasts were built to examine results for each condition against the baseline, followed by a contrast of simple tasks against the corresponding complex conditions. An Omnibus F-contrast was built to examine activity in the SC from all conditions together. We analyzed within-subject GLM contrast estimates at the VOI mentioned above. We expected positive signal increases and considered results significant if the lower bound of the two-sided 90% confidence interval, which amounted to 95% confidence for our uni-directional hypothesis, did not include the zero baseline. Additionally, we conducted a voxel-wise analysis of cortical regions and SC surrounding the VOI. Results were considered significant, surviving a voxel-level threshold of 0.05 FWE-corrected for cortical results and 0.001 uncorrected for results in the SC.

We extracted raw time courses spanning all sessions from each subject to examine voxel signal time courses. The raw time courses were then de-meaned across sessions, regressed with motion parameters, and high-pass filtered at 128 Hz. The time courses were interpolated to 0.1 s and later re-sampled to 2 s intervals aligned to the start of each experimental block. The time courses were then separated by conditions using their respective block onsets. A within-subject mean time course with standard errors was computed for each condition.

## Data Availability

The datasets used and generated during the current study are available from the corresponding author on reasonable request.
